# The miR-204-5p/FOXC1/GDF7 axis regulates the osteogenic differentiation of human adipose-derived stem cells via the AKT and p38 signalling pathways

**DOI:** 10.1186/s13287-020-02117-4

**Published:** 2021-01-18

**Authors:** You Zhou, Siyu Liu, Wei Wang, Qiang Sun, Mengzhu Lv, Shude Yang, Shuang Tong, Shu Guo

**Affiliations:** 1 Department of Plastic Surgery, The First Hospital of China Medical University, NO 155 Nanjing street Heping Strict, Shenyang, 110001 Liaoning Province China; 2 Institute of Respiratory Disease, The First Hospital of China Medical University, NO 155 Nanjing street Heping Strict, Shenyang, 110001 Liaoning Province China

**Keywords:** Human adipose-derived stem cells, Osteogenic differentiation, miR-204-5p, Forkhead box C1, Growth differentiation factor 7

## Abstract

**Background:**

Human adipose-derived stem cells (hADSCs) are stem cells with the potential to differentiate in multiple directions. miR-204-5p is expressed at low levels during the osteogenic differentiation of hADSCs, and its specific regulatory mechanism remains unclear. Here, we aimed to explore the function and possible molecular mechanism of miR-204-5p in the osteogenic differentiation of hADSCs.

**Methods:**

The expression patterns of miR-204-5p, *Runx2*, alkaline phosphatase (*ALP*), osteocalcin (*OCN*), forkhead box C1 (*FOXC1*) and growth differentiation factor 7 (*GDF7*) in hADSCs during osteogenesis were detected by qRT-PCR. Then, ALP and alizarin red staining (ARS) were used to detect osteoblast activities and mineral deposition. Western blotting was conducted to confirm the protein levels. The regulatory relationship among miR-204-5p, FOXC1 and GDF7 was verified by dual-luciferase activity and chromatin immunoprecipitation (ChIP) assays.

**Results:**

miR-204-5p expression was downregulated in hADSC osteogenesis, and overexpression of miR-204-5p suppressed osteogenic differentiation. Furthermore, the levels of FOXC1 and GDF7 were decreased in the miR-204-5p mimics group, which indicates that miR-204-5p overexpression suppresses the expression of FOXC1 and GDF7 by binding to their 3′-untranslated regions (UTRs). Overexpression of FOXC1 or GDF7 improved the inhibition of osteogenic differentiation of hADSCs induced by the miR-204-5p mimics. Moreover, FOXC1 was found to bind to the promoter of miR-204-5p and GDF7, promote the deacetylation of miR-204-5p and reduce the expression of miR-204-5p, thus promoting the expression of GDF7 during osteogenic differentiation. GDF7 induced hADSC osteogenesis differentiation by activating the AKT and P38 signalling pathways.

**Conclusions:**

Our results demonstrated that the miR-204-5p/FOXC1/GDF7 axis regulates the osteogenic differentiation of hADSCs via the AKT and p38 signalling pathways. This study further revealed the regulatory mechanism of hADSC differentiation from the perspective of miRNA regulation.

## Introduction

Bone tissue engineering combines seed cells with biomaterials under the action of osteogenic inducing factors to differentiate seed cells into osteoblasts and achieve bone regeneration [1]. Therefore, seed cells are the primary link and basic elements of bone tissue engineering. hADSCs are some of the most widely used seed cells in bone tissue engineering [2]. Zuk et al. [3] first isolated hADSCs from human adipose tissue. hADSCs can be conveniently accessed by aspirating adipose tissue. Gronthos et al. have shown that resting stage hADSCs constitute 70% of the total number of cells with stem cell properties, and existing research has shown that hADSCs have equal multi-directional differentiation potential. They can differentiate into adipocytes, cardiomyocytes, nerve cells, osteoblasts, chondrocytes and so on [4]. hADSCs have many biological characteristics, such as self-renewal, multi-directional differentiation, specific migration to the injury site, and functions for tissue repair and regeneration [5]. These advantages make them favoured for clinical application, but the mechanisms of the osteogenic differentiation of hADSCs are far less understood.

MicroRNAs are a class of small molecule RNAs, approximately 22 nt in length, that are endogenously expressed in eukaryotes [6]. They can inhibit target gene transcription or mRNA degradation by binding to the target gene [7]. MicroRNAs are involved in the regulation of a variety of biological processes, including cell proliferation and differentiation, metabolic apoptosis, DNA damage repair, cancer development, embryonic development, tissue differentiation and formation, and other physiological processes [8, 9]. Studies have reported that microRNAs can regulate the osteogenic differentiation of mesenchymal stem cells (MSCs) [10, 11], and to the best of our knowledge, at least 27 microRNAs have been reported to participate in the osteogenic differentiation of ADSCs. miR-20a has been reported to play a positive role in regulating the osteogenic differentiation of hADSCs by co-regulating the BMP signalling pathway, while miR-23a inhibits osteogenic differentiation by acting on the key transcription factor Runx2 [12, 13]. miR-204-5p is a member of the miR-204 family, which is derived from the 5′ end arm of the hsa-miR-204 precursor (pre-miRNA) [14]. Studies have shown that miR-204 is significantly downregulated during osteogenic differentiation and inhibits osteogenic differentiation by downregulating the important transcription factor Runx2 during osteogenic differentiation [15]. Nevertheless, the specific mechanism by which miR-204 regulates the osteogenic differentiation of hADSCs remains largely unknown. Moreover, complex feedback regulation exists between these miRNAs and their target osteogenic genes [11], so the regulation of the expression and degradation of these miRNAs in the differentiation process of hADSCs is worth further study.

Forkhead box C1 (FOXCl), a member of the fox superfamily, is widely present in various tissues and organs and regulates biological processes, including cell differentiation, embryonic development, tumorigenesis and development [16]. During embryonic development, FOXC1 activates target gene transcription by binding to the promoter of the target gene or interacting with other transcription factors [17]. FOXC1 is an important regulator of the normal development and formation of bones originating from both endochondral and intramembranous origins [18]. The Foxc1^−/−^ mutant mice showed a large number of abnormalities in the skulls, vertebrae, ribs and appendage bones [19, 20]. A recent analysis showed that the ossification centres in the long bones of Foxc1^−/−^ mutant mice were reduced, and Foxc1 regulated the PTHrP expression through interactions with Indian Hedgehog (Ihh)-Gli2 signalling, suggesting that FOXC1 played a role in regulating chondrocyte proliferation and differentiation [21]. Growth differentiation factor 7 (GDF7), also known as bone morphogenetic protein 12 (BMP12) and cartilage-derived morphogenetic protein 3 (CDMP3), is a member of the transforming growth factor beta (TGFβ) superfamily [22, 23]. Shen et al. found that after GDF7 stimulated ADSCs, it could promote the osteogenic differentiation of ADSCs through the Smad1/5/8 pathway [24]. However, the roles of FOXC1 and GDF7 in the osteogenic differentiation of hADSCs are still unclear, and the mechanisms also need to be further investigated.

Studies have reported that GDF7 and FOXC1 are highly expressed during hADSC osteogenic differentiation, and miR-204-5p is expressed at low levels during osteogenic differentiation [25, 26]. Moreover, TargetScan/miRDB predicts that both *GDF7* and *FOXC1* might be the target genes of miR-204-5p, and the JASPAR software shows that the transcription factor FOXC1 is able to bind to the promoter of miR-204-5p and *GDF7*. Therefore, we speculated that miR-204-5p might act on the mRNA of *GDF7* and *FOXC1* to inhibit the osteogenic differentiation of hADSCs, and FOXC1 might bind to the promoters of miR-204-5p and *GDF7* and reduce the expression of miR-204-5p, thus promoting the expression of *GDF7*. In this study, we aimed to investigate the role of the miR-204-5p/FOXC1/GDF7 regulatory axis in hADSC osteogenic differentiation. Our data indicated that the miR-204-5p/FOXC1/GDF7 axis formed cyclic regulation, which plays an important role in the balance of osteogenic differentiation. To our knowledge, the relationship among miR-204-5p, FOXC1 and GDF7 has not been studied during the osteogenic differentiation of hADSCs. Our data are expected to provide new insight into the differentiation of hADSCs in bone regeneration and promote hADSC clinical application.

## Materials and methods

### Cell culture and osteogenic induction

hADSCs used in this study were all derived from discarded adipose tissue after liposuction from patients in the Department of Plastic Surgery, The First Hospital of China Medical University. The samples were from 8 female patients aged 29.56 ± 5.85 years without metabolic disease, syphilis, HIV, hepatitis or other systemic diseases. The surgical site was the abdomen, and the surgical method was suction. The above experimental samples were collected with the informed consent of the clinical patients and approved by the Ethics Committee of the First Hospital of China Medical University.

The adipose tissue obtained by liposuction of subcutaneous adipose tissue was added into 50-mL centrifuge tubes and washed with sterile phosphate-buffered saline (PBS; Gibco, Grand Island, NY, USA). Then, the samples were centrifuged at 1000*g* for 5 min to remove residual blood cells and tissue debris. The above procedure was repeated 3–4 times before enzymatic digestion with 0.2% collagenase type I (Sigma-Aldrich, St. Louis, MO, USA) at 37 °C for 45 min. Dulbecco’s modified Eagle’s medium/Nutrient F-12 Ham (DMEM/F12 (HyClone, USA)), containing 10% foetal bovine serum (FBS (Gibco, USA)), was added to the digested lipoaspirates for 5 min to neutralize enzyme activity, followed by centrifugation at 1000*g* for 5 min. Finally, the cells were plated in 75-cm^2^ culture flasks and incubated in a culture medium (DMEM/F12, 10% FBS, 1% penicillin-streptomycin solution (Gibco, USA)) at 37 °C in 5% CO_2_ with saturated humidity. The medium was changed for the first time after 24 h and then every 2 days after. hADSCs were passaged until they were 90% confluent; 0.25% trypsin:0.2% EDTA at a ratio of 1:3 was used to dissociate the cells. hADSCs at passage 3 were used for subsequent experiments. When the cells adhered to the wall and grew to approximately 90% confluence, they were cultured in groups with proliferation medium (PM) or complete OriCell osteogenic differentiation medium (Cyagen, USA) (OM).

### Flow cytometric analysis

When hADSCs grew to 80% confluence, the old culture medium was removed, and the cells were washed once or twice with PBS. Trypsin solution (Gibco, USA) was added, and the cells were placed in an incubator at 37 °C for 1 min and observed under an inverted microscope. The cells separated and presented granular circles with the addition of the complete medium of fresh stem cells and were blown evenly with a pipette to prepare a single-cell suspension. The single-cell suspension was transferred to a flow tube, washed with PBS twice and centrifuged at 1000 rpm for 5 min. The supernatant was discarded, and the cells were resuspended with 500 μL 1× binding buffer and incubated with CD44, CD45, CD90, CD105 and HLA-DR antibody (all at 1:50 dilution) working solution (Abcam, Cambridge, MA, USA) in each group, and the homotype control group was set at the same time. After incubation, the cells were centrifuged at 1000*g* for 5 min. The supernatant was discarded, and the cells were resuspended in 500 μL 1× buffer and analysed by flow cytometry with a FACSCalibur flow cytometer (Becton Dickinson, Mountain View, CA, USA). The data were analysed using the CELL Quest software.

### RNA oligoribonucleotides and plasmid and transfection

The RNA oligoribonucleotides used in this study, including miR-204-5p mimics, miR-204-5p inhibitor, the small interfering RNAs (siRNAs) targeting FOXC1 (si-FOXC1) or GDF7 (si-GDF7), and the miR negative control (miR-NC) and siRNA control (si-NC), were purchased from GenePharma Co. (Shanghai, China). The FOCX1 and GDF7 plasmids and the empty vector were provided by Vipotion (Guangzhou, China).

At the time of passage, the surface of the culture vessel was covered with gelatine: an appropriate amount of 0.1% gelatine was added to the culture vessel to cover the whole bottom of the culture plate. The culture vessel was placed on a super clean table, and the gelatine was discarded after 30 min. After the culture vessel was dried, it could be used for cell inoculation. hADSCs were inoculated onto gelatine-coated plates and cultured in a culture medium containing 10% FBS. For transfection, when hADSCs reached 70–90% confluence, they were transfected with mimics (100 nM) or inhibitor (100 nM) or with the FOXC1 or GDF7 plasmids or empty vector (20 ng) or siRNA (20 μM) using Lipofectamine 3000 (Invitrogen, Carlsbad, CA, USA) according to the manufacturer’s procedure. Cells were cultured in PM or OM for 14 days. The old medium was discarded every 3 days, the transfection processes were repeated according to the above steps and the medium was replaced with fresh complete medium. The cells were harvested at 14 days for RNA and protein analysis.

### RNA extraction and quantitative polymerase chain reaction (qPCR)

Total cellular RNA was isolated at 14 days or each time point after osteoinduction or normal culturation using TRIzol Reagent (Invitrogen) according to the manufacturer’s instructions. Briefly, the extracted RNA was reverse-transcribed in the presence of a poly-A polymerase with an oligo-dT adaptor. Gene expression levels were measured by the real-time PCR detection system (Bio-Rad, Hercules, CA, USA) by SYBR Green (Bio-Rad, Hercules, CA, USA) detection. The expression of miR-204-5p, Runx2, ALP, OCN, FOXC1 and GDF7 was quantified by qPCR using SYBR Green assays (TaKaRa). GAPDH and U6 were used as internal controls for mRNAs and miR-204-5p. The data were calculated using the 2^−ΔΔCt^, where RQ is the relative quantity, expressed as the fold change relative to the gene expression levels in the control samples. The primer sequences used in qPCR were as follows: miR-204-5p-forward 5′-ACACTCCAGCTGGGTTCCCTTTGTCATCCTAT-3′, miR-204-5p-reverse 5′-CTCAACTGGTGTCGTGGAGTCGGCAATTCAGTTGAGAGGCATAG-3′; *Runx2*-forward 5′-TGGTTACTGTCATGGCGGGTA-3′, *Runx2*-reverse 5′-TCTCAGATCGTTGAACCTTGCTA-3′ (101 bp, GenBank accession no. NM_001015051); *ALP*-forward 5′-ACTGGTACTCAGACAACGAGAT-3′, *ALP*-reverse 5′-ACGTCAATGTCCCTGATGTTATG-3′ (97 bp, GenBank accession no. NM_003064); *OCN*-forward 5′-CACTCCTCGCCCTATTGGC-3′, *OCN*-reverse 5′-CCCTCCTGCTTGGACACAAAG-3′ (112 bp, GenBank accession no. NM_199173); *FOXC1*-forward 5′-TGTTCGAGTCACAGAGGATCG-3′, *FOXC1*-reverse 5′-ACAGTCGTAGACGAAAGCTCC-3′ (122 bp, GenBank accession no. NM_001453); *GDF7*-forward 5′-TGATGTCGCTTTACCGGAGC-3, *GDF7*-reverse 5′-CTGCCGATTCGTCTTGGGT-3′ (129 bp, GenBank accession no. AB158468); *GAPDH*-forward 5′-TGTTCGTCATGGGTGTGAAC-3′, *GAPDH*-reverse 5′-ATGGCATGGACTGTGGTCAT-3′ (154 bp, GenBank accession no. AF261085); and *U6*-forward 5′-CTCGCTTCGGCAGCACA-3′, *U6*-reverse 5′-AACGCTTCACGAATTTGCGT-3′ (96 bp, GenBank accession no. NR_004394).

### Western blot analysis

After 14 days of osteoinduction or normal culture, the proteins of hADSCs were extracted by using RIPA buffer containing 1% PMSF (Sigma-Aldrich). The BCATM Protein Assay Kit (Pierce, Appleton, WI, USA) was used for quantification of protein samples. Equal amounts of protein samples (30 μg) were separated on 12% SDS-PAGE gels and transferred to PVDF membranes (Millipore Corporation, Billerica, MA, USA). After blocking in 0.5% bovine serum albumin (BSA; Roche) for 1 h at 37 °C, the blocked membranes were incubated with the corresponding primary antibodies overnight at 4 °C, including FOXC1 (ab223850, 1:1000), ALP (ab16695, 1:1000), RUNX2 (ab23981, 1 μg/ml), OCN (ab93876, 1 μg/ml), P38 (ab170099, 1:1000), p-P38 (ab4822, 1:500), AKT (ab179463, 1:1000), p-AKT (ab38449, 1:500) and GAPDH (ab181602, 1:1000), which were purchased from Abcam (Cambridge, MA, USA). The membranes were then washed and incubated with appropriate HRP secondary antibodies (ab6721, 1:2000) (Cambridge, MA, USA) for 1 h at 37 °C. The signals were detected and analysed by an enhanced chemiluminescence system (Amersham Biosciences, Piscataway, NJ, USA) and Image Lab software (Bio-Rad, Cal, USA). Protein levels were quantified using Image Lab software.

### Alkaline phosphatase (ALP) staining

Transfected hADSCs cultured in PM or OM for 14 days were assayed for ALP staining and activity. The medium was removed after 14 days, and the cells were washed twice with PBS and fixed with 4% paraformaldehyde (Jianglaibio, Shanghai, China) for 30 min. The paraformaldehyde was removed, and the cells were washed with ddH2O three times, and alkaline phosphatase staining solution (Beyotime, China) was added for 30 min. The ALP staining solution was removed, and the cells were washed with ddH2O three times and observed under a microscope (Leica DMIRB, Germany) and imaged.

### Alizarin red staining (ARS)

Transfected hADSCs cultured in PM or OM for 14 days were assayed for ARS. The culture medium was discarded, and the cells were fixed with 4% paraformaldehyde for 15–20 min and washed with PBS 3 times. ARS solution (ScienCell, San Diego, CA, USA) was prepared in advance and added to the culture plate; the plate was placed in the incubator for 15 min, the staining solution was removed and the sample was washed with PBS solution three times; the PBS was removed, and the samples were placed under a differential microscope to take photos (Leica DMIRB, Germany).

### Immunofluorescence staining

hADSCs transiently transfected with miR-204-5p mimics or inhibitors were seeded in 6-well plates. After 14 days in PM or OM, cells grown on sterile glass coverslips were fixed in 4% paraformaldehyde (Jianglaibio, Shanghai, China) for 30 min, permeabilized with 0.1% Triton X-100 for 15 min and blocked with 5% normal goat serum for 30 min. Then, cells were incubated with a primary antibody-OCN (ab13418, 1:500; Abcam) and incubated with an anti-mouse secondary antibody (ab150117, 1:500; Abcam) for 1 h at room temperature. 4,6-Diamidino-2-phenylindole (DAPI) was used to stain the nuclei, and the coverslips were mounted on a glass slide and observed under a confocal Zeiss Axiovert 650 microscope at 488 nm (green, OCN) and 405 nm (blue, DAPI). Images were captured using an LSM 5 Exciter confocal imaging system (Carl Zeiss).

### Dual-luciferase reporter assay

Luciferase reporter assays were carried out as follows. First, the 3′-UTR sequences of wild-type (WT) and mutant-type (MUT) FOXC1 and GDF7 were cloned into the psiCHECk2 vector, while the WT and MUT regions of the promoters of miR-204-5p and GDF7 were cloned into the pGL3 vector. To analyse the interaction between miR-204-5p and FOXC1 or GDF7, hADSCs (1 × 10^5^) were grown in a 96-well plate and co-transfected with either miR-204-5p mimics (100 nM) or NC mimics (100 nM), FOXC1-WT/MUT (20 ng), or GDF7-WT/MUT (20 ng) by Lipofectamine 2000 (Invitrogen). To assess FOXC1 binding to the promoter of GDF7 and miR-204-5p, hADSCs were grown in a 96-well plate and co-transfected with either vector/vector-FOXC1 or miR-204-5p-WT/MUT or GDF7-WT2/MUT2. Renilla and firefly luciferase activities were measured 48 h after transfection using the Dual-Luciferase Reporter Assay System (Promega, Beijing, China). All luciferase values were normalized to those of firefly luciferase and expressed as fold induction relative to the basal activity.

### Chromatin immunoprecipitation (ChIP) assay

ChIP assays were conducted by using an EZ-Magna ChIP assay kit (Merck Millipore). hADSCs were seeded in 10-cm dishes and transfected with FOXC1 or the vector. The cells were crosslinked with 1% formaldehyde and sonicated to shear DNA. Then, the DNA-protein complexes were isolated with antibodies against isotype immunoglobulin G (IgG) and HDAC2, H3K9AC, and FOXC1 (Cell Signaling). The protein-DNA complexes were then purified and reverse-crosslinked. The DNA was isolated and quantified by qRT-PCR. Relative enrichment was calculated as the amount of amplified DNA relative to values obtained from Input. The primer sequences used in ChIP assays were as follows: FOXC1-miR-204-5p-forward 5′-TGGGGTAGTTGCCAGTTAGA-3′, FOXC1-miR-204-5p-reverse 5′-TCTGATGTGGTTGAATGTCAGA-3′; FOXC1-GDF7-forward 5′-AAACACCCAAACACTGCGG-3′, FOXC1-GDF7-reverse 5′-GGGATAGTCCACCCTGCTTCT-3′; H3K9ac-miR-204-5p-forward 5′-ACCACAGAAGTCTTCATTTCCT-3′, H3K9ac-miR-204-5p-reverse 5′-AATAGTGCCGTCAAGCTGTC-3′; and HDAC2-miR-204-5p-forward 5′-GAAGGGCTGGATGATGCTCT-3′, HDAC2-miR-204-5p-reverse 5′-GCAGATGGATTACCCAATTTACAT-3′.

### Statistical analysis

All the corresponding experiments were independently repeated more than three times, and the Western blot results are representative. All data in this experiment are expressed as the mean ± standard deviation. SPSS 17.0 for Windows statistical software was used for the Kruskal-Wallis one-way analysis of variance (ANOVA) test, and *P* < 0.05 indicated a significant difference.

## Results

### miR-204-5p is downregulated during the osteogenic differentiation of hADSCs

**Fig. 1:**
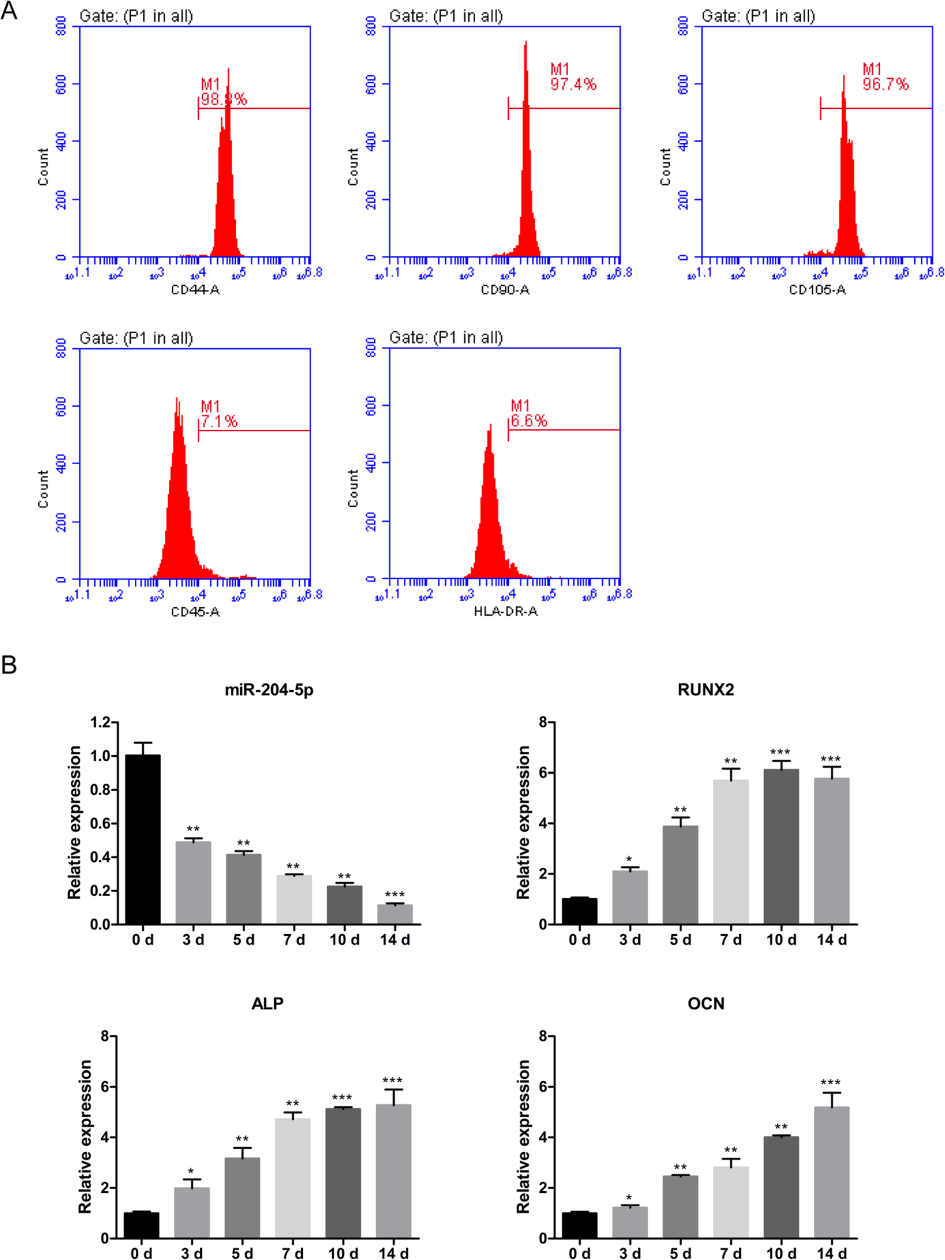
miR-204-5p is downregulated during the osteogenic differentiation of hADSCs. **a** Flow cytometry was used to identify hADSCs by detecting specific marker proteins CD44, CD45, CD90, CD105 and HLA-DR. **b** Relative expression of miR-204-5p at each time point during the osteogenic differentiation of hADSCs detected by qRT-PCR. U6 was used as a reference gene. Relative mRNA levels of the osteogenic markers RUNX2, ALP and OCN at each time point during the osteogenic differentiation of hADSCs detected by qRT-PCR. GAPDH was used as an internal control gene. Data are presented as the mean ± SD. *p < 0.05, **p < 0.01, ***p < 0.01 (*n* = 3 independent experiments)

As a mesenchymal lineage cell population, ADSCs express CD44, CD90 and CD105, while haematopoietic markers such as CD45 and histocompatibility leukocyte antigen (HLA)-DR are absent. hADSCs were collected and phenotypically identified, and the results showed that the cells were positive for CD44, CD90 and CD105 and negative for CD45 and HLA-DR, which suggested that the primary cultured cells were hADSCs (Fig. 1a). These identified hADSCs were cultured under osteogenic induction conditions for 14 days. Since it has been reported that miR-204 is downregulated during osteogenic differentiation, we detected the expression level of hsa-miR-204-5p by qRT-PCR at 3, 5, 7, 10 and 14 days in these identified hADSCs. Consistent with a previous report, the results showed that the expression level of miR-204-5p decreased gradually with induction time (Fig. 1b). At the same time, we detected the expression levels of the osteogenic markers *RUNX2*, *ALP* and *OCN* in cells at various time points. The qRT-PCR results showed that the expression levels of *RUNX2*, *ALP* and *OCN* were markedly upregulated during osteogenesis (Fig. 1b).

### miR-204-5p inhibits the osteogenic differentiation of hADSCs in vitro

**Fig. 2:**
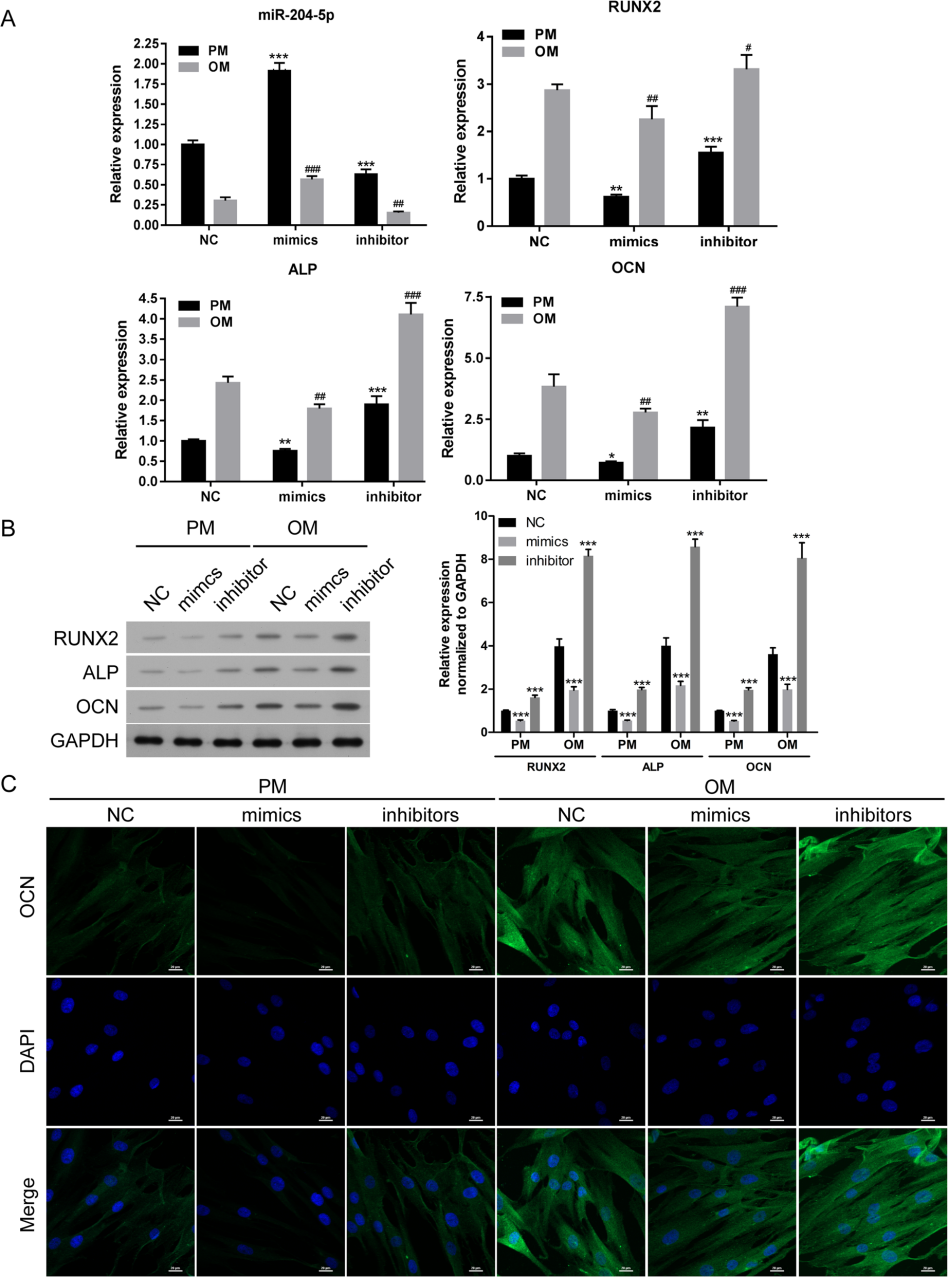
miR-204-5p overexpression inhibits the expression of RUNX2, ALP and OCN. hADSCs were transfected with miR-204-5p mimics, miR-204-5p inhibitors or NC and cultured in PM or OM. **a** Relative mRNA levels of miR-204-5p, RUNX2, ALP and OCN measured by qRT-PCR on day 14 of osteogenic induction. U6 and GAPDH were used as internal control genes. The data were normalized to the NC-PM group. **b** Left panel: Western blot of RUNX2, ALP and OCN protein levels on day 14 after osteogenic induction. GAPDH was used as an internal control. Right panel: quantitative analysis of the protein levels. **c** Confocal microscopy of cells with OCN with DAPI counterstaining on day 14 after osteogenic induction. Scale bars, 20 μm. Data are presented as the mean ± SD. *hADSCs transfected with miR-204-5p mimics or inhibitors vs NC in PM; ^#^hADSCs transfected with miR-204-5p mimics or inhibitors vs NC in OM. * or ^#^*p* < 0.05, ** or ^##^*p* < 0.01, *** or ^###^*p* < 0.001 (*n* = 3 independent experiments)

To explore the effect of miR-204-5p on the osteogenic differentiation of hADSCs, we transfected miR-204-5p mimics or inhibitor into hADSCs and cultured them in PM or OM. The expression level of miR-204-5p was detected by qRT-PCR at 14 days in PM and OM, and it was increased when cells were transfected with miR-204-5p mimics but decreased when cells were transfected with miR-204-5p inhibitor (Fig. 2a). qRT-PCR and Western blotting were used to detect the mRNA and protein expression levels of the osteogenic markers RUNX2, ALP and OCN. The results showed that miR-204-5p mimics could decrease RUNX2, ALP and OCN expression, whereas miR-204-5p inhibitor could increase RUNX2, ALP and OCN expression (Fig. 2a, b). In addition, immunofluorescence staining indicated that the protein level of OCN was reduced in the miR-204-5p overexpression group and increased in the miR-204-5p inhibition group (Fig. 2c).

**Fig. 3:**
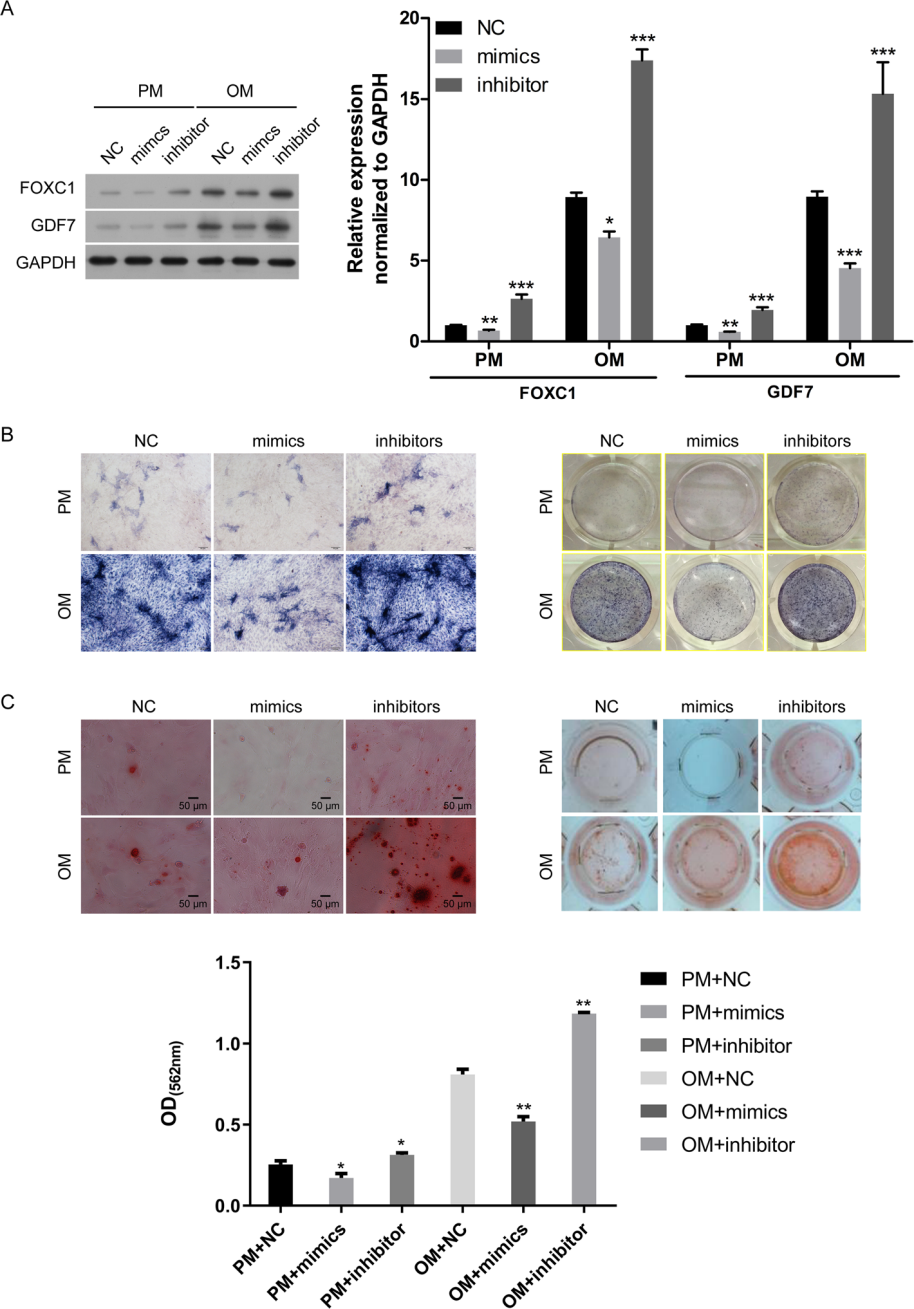
miR-204-5p overexpression inhibits the osteogenic differentiation of hADSCs. hADSCs were transfected with miR-204-5p mimics, miR-204-5p inhibitors or NC and cultured in PM or OM. **a** Left panel: Western blot of FOXC1 and GDF7 protein levels on day 14 after osteogenic induction. GAPDH was used as an internal control. Right panel: quantitative analysis of the protein levels. ALP staining (**b**) and ARS (**c**) on day 14 after osteogenic induction. Quantification of ARS was shown below. Data are presented as the mean ± SD. *p < 0.05, **p < 0.01, ***p < 0.001 (*n* = 3 independent experiments)

Moreover, Western blot analysis showed that the protein levels of FOXC1 and GDF7 were also decreased in the miR-204-5p mimic group and increased in the miR-204-5p inhibition group (Fig. 3a).

ALP staining showed that miR-204-5p mimics suppressed the osteogenic differentiation of hADSCs, and miR-204-5p inhibitor enhanced the osteogenic differentiation of hADSCs in PM or OM (Fig. 3b). The extracellular mineralization of hADSCs was measured by ARS assay in PM or OM on day 14, and the results showed that the osteogenic differentiation of hADSCs was suppressed with miR-204-5p mimics but increased with miR-204-5p inhibitor (Fig. 3c). Taken together, these results suggested that miR-204-5p inhibits the osteogenic differentiation of hADSCs in vitro.

### FOXC1 reduces the suppressive effect of miR-204-5p on the osteogenic differentiation of hADSCs

**Fig. 4:**
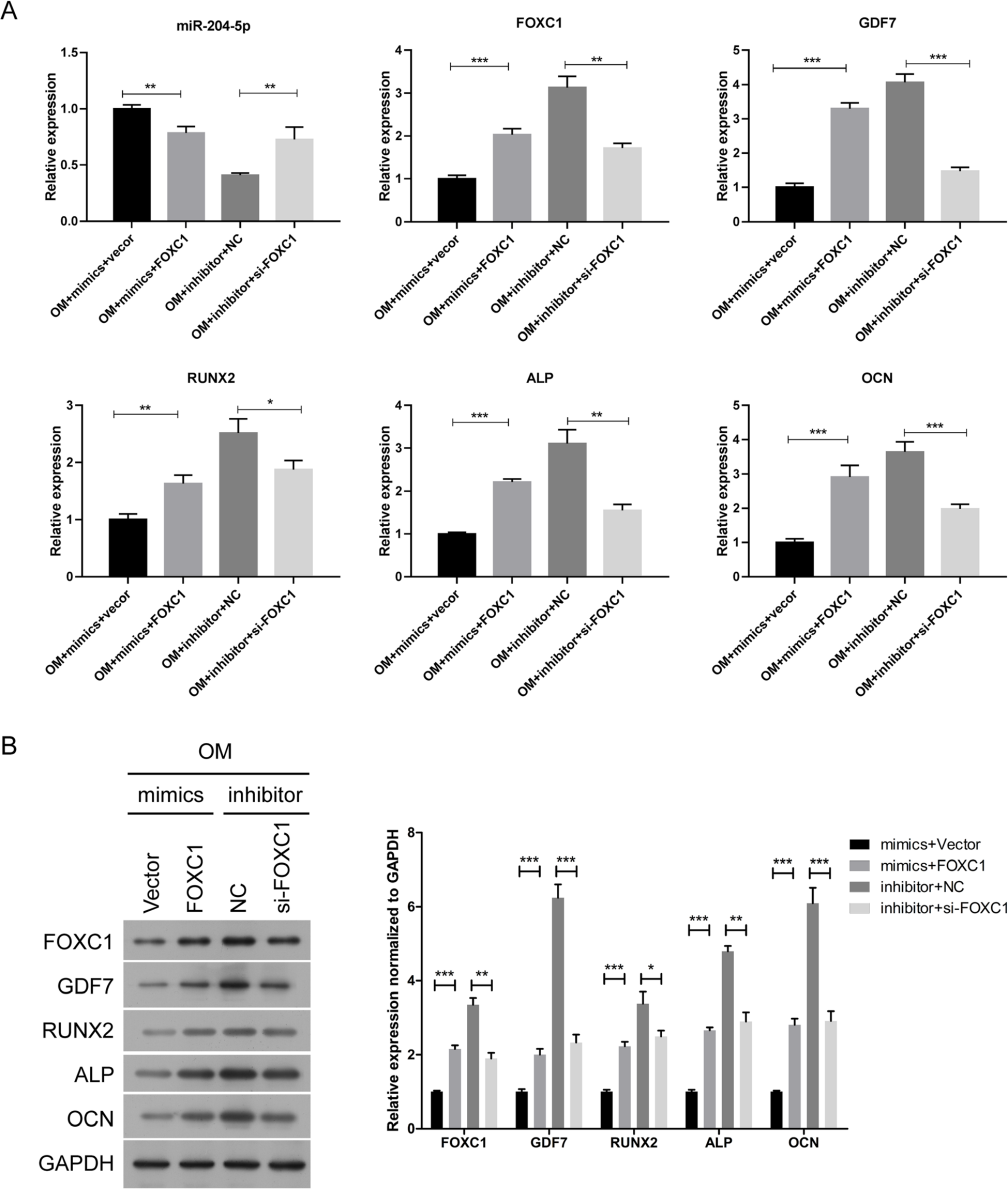
FOXC1 reduces the suppressive effect of miR-204-5p on the expression of GDF7, RUNX2, ALP and OCN. hADSCs were co-transfected with miR-204-5p mimics and FOXC1 overexpression vector or empty vector or co-transfected with miR-204-5p inhibitors and si-FOXC1 and cultured in OM. **a** Relative expression of miR-204-5p, FOXC1, GDF7, RUNX2, ALP and OCN measured by qRT-PCR on day 14 of osteogenic induction. U6 and GAPDH were used as internal control genes. **b** Left panel: Western blot of FOXC1, GDF7, RUNX2, ALP and OCN protein levels on day 14 after osteogenic induction. GAPDH was used as an internal control. Right panel: quantitative analysis of the protein levels. Data are presented as the mean ± SD. *p < 0.05, **p < 0.01, ***p < 0.001 (*n* = 3 independent experiments)

**Fig. 5:**
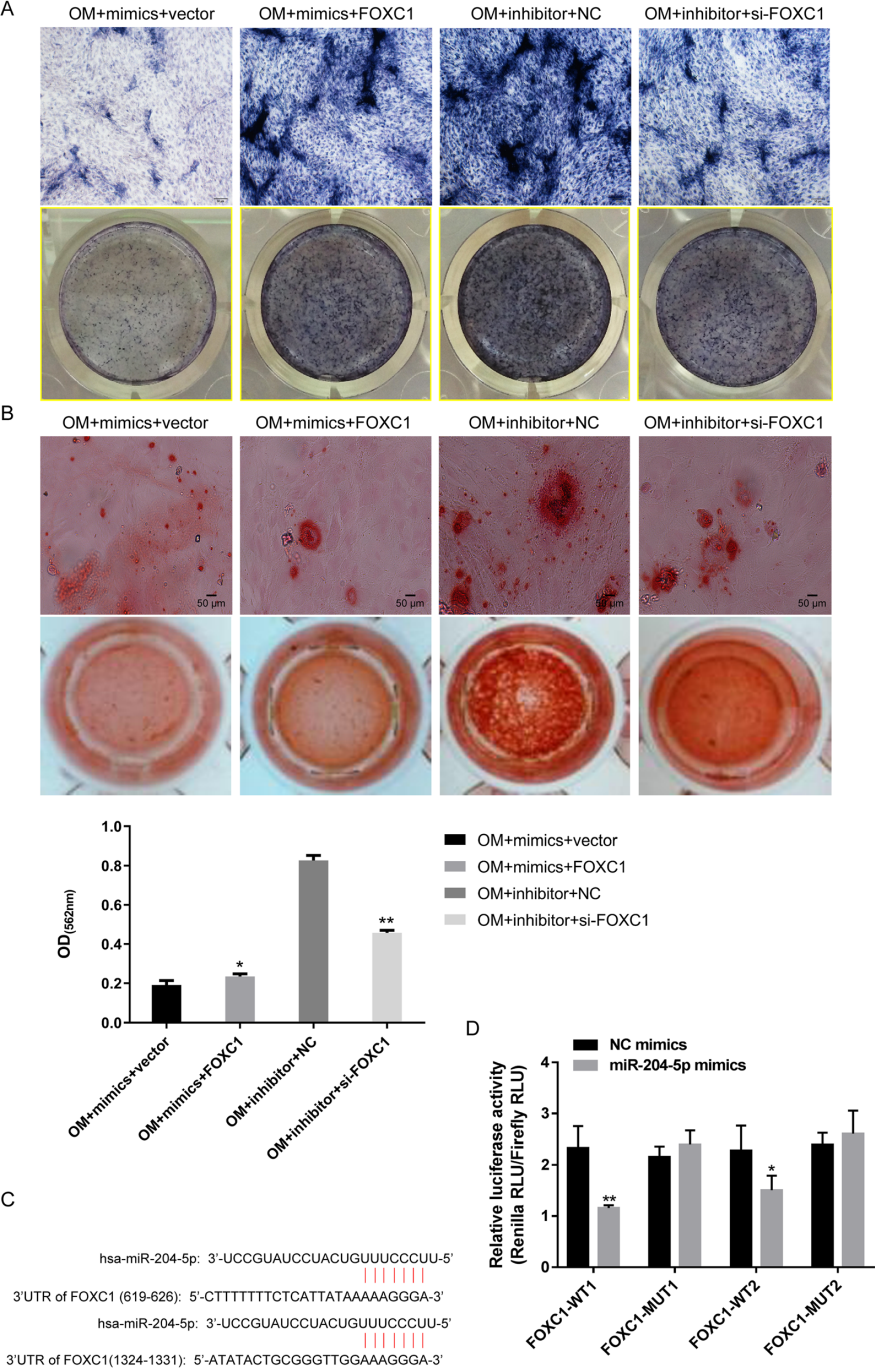
FOXC1 reduces the suppressive effect of miR-204-5p on the osteogenic differentiation of hADSCs. hADSCs were co-transfected with miR-204-5p mimics and FOXC1 overexpression vector or empty vector or co-transfected with miR-204-5p inhibitors and si-FOXC1 and cultured in OM. ALP staining (**a**) and ARS (**b**) on day 14 after osteogenic induction. Quantification of ARS was shown below. **c** Predicted binding site of miR-204-5p in the 3′-UTR of FOXC1 mRNA. **d** Relative luciferase activity of cells in different groups. Data are presented as the mean ± SD. *p < 0.05, **p < 0.01, ***p < 0.001 (*n* = 3 independent experiments)

To investigate the role of FOXC1 in osteogenic differentiation, the cells were co-transfected with miR-204-5p mimics and FOXC1 plasmid or co-transfected with miR-204-5p inhibitor and FOXC1 siRNA. Compared with the empty vector transfection, FOXC1 overexpression inhibited miR-204-5p expression but promoted the expression of *FOXC1* and *GDF7* and increased *RUNX2*, *ALP* and *OCN* expression under miR-204-5p mimic transfection, whereas FOXC1 downregulation obtained the opposite results in hADSCs transfected with miR-204-5p inhibitor (Fig. 4a). Then, we also carried out Western blotting to evaluate the protein expression levels of FOXC1, GDF7, RUNX2, ALP and OCN and obtained the same results as with qRT-PCR (Fig. 4b). Subsequently, ALP staining and ARS showed that overexpression of FOXC1 attenuated the suppression effect of miR-204-5p on the osteogenic differentiation and extracellular mineralization of hADSCs, while downregulation of FOXC1 inhibited the osteogenic differentiation and extracellular mineralization of hADSCs induced by the miR-204-5p inhibitor (Fig. 5a, b). These results suggested that FOXC1 reduces the suppressive effect of miR-204-5p on the osteogenic differentiation of hADSCs.

To explore the relationship between miR-204-5p and FOXC1, we used TargetScan/miRDB to search for potential targets of miR-204-5p. We found that the 3′-UTR of *FOXC1* has two miR-204-5p binding sites (Fig. 5c). Next, we constructed a WT reporter and MUT reporter that contained a mutant 3′-UTR of *FOXC1* designed with mutated sequences of the miR-204-5p binding site. The results indicated that overexpression of miR-204-5p markedly decreased the luciferase activity in the WT group of FOXC1 cells, whereas this phenomenon was not observed in the MUT group (Fig. 5d), which suggested that FOXC1 is a target of miR-204-5p.

### GDF7 impairs the suppressive effect of miR-204-5p on the osteogenic differentiation of hADSCs

**Fig. 6:**
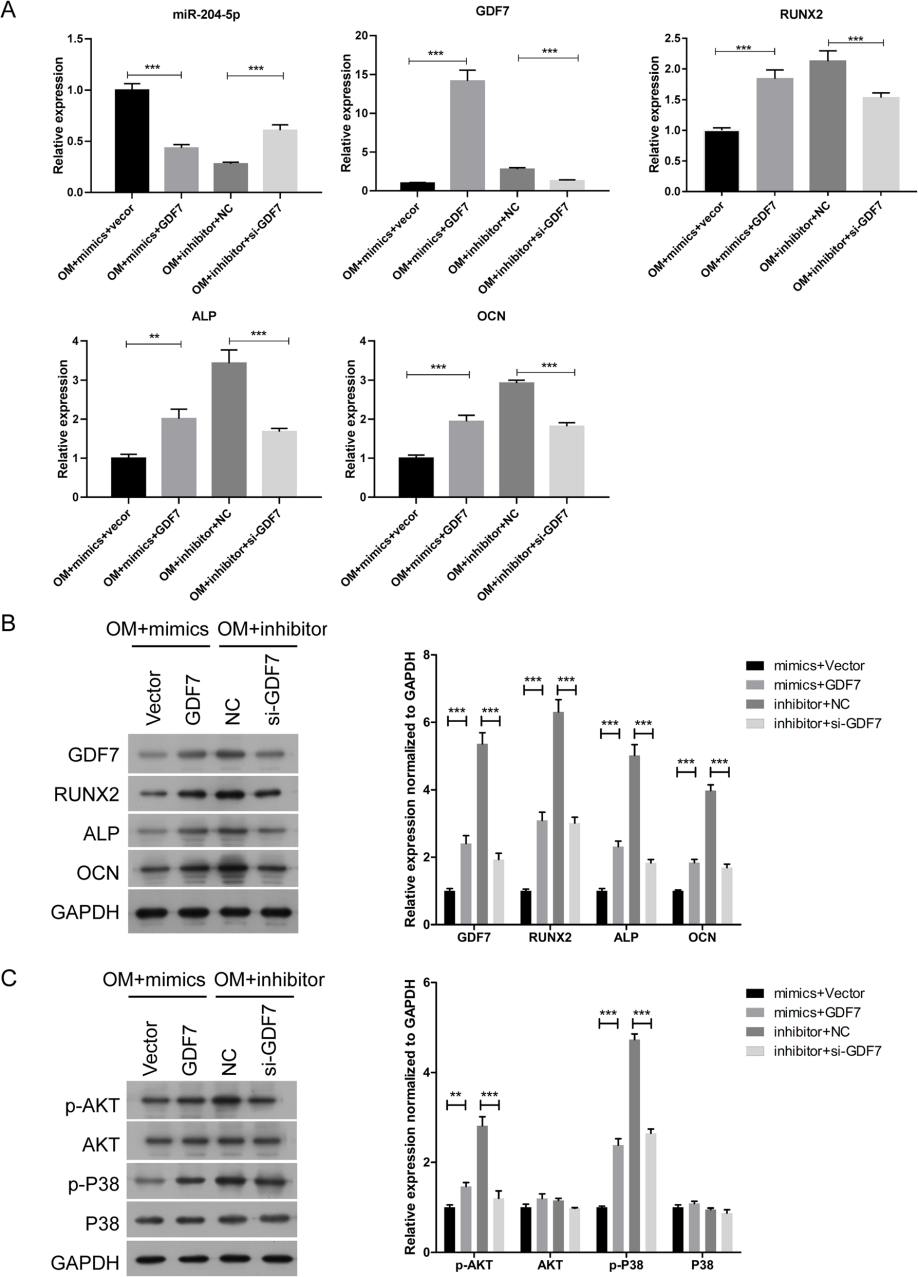
GDF7 impairs the suppressive effect of miR-204-5p on the expression of RUNX2, ALP, OCN, p-AKT and p-P38. hADSCs were co-transfected with miR-204-5p mimics and GDF7-overexpression vector or empty vector or co-transfected with miR-204-5p inhibitors and si-GDF and cultured in OM. **a** Relative expression of miR-204-5p, GDF7, RUNX2, ALP and OCN measured by qRT-PCR on day 14 of osteogenic induction. U6 and GAPDH were used as internal control genes. **b** Left panel: Western blot of GDF7, RUNX2, ALP and OCN protein levels on day 14 after osteogenic induction. GAPDH was used as an internal control. Right panel: quantitative analysis of the protein levels. **c** Left panel: Western blot of p-AKT, AKT, p-P38 and P38 protein levels on day 14 after osteogenic induction. GAPDH was used as an internal control. Right panel: quantitative analysis of the protein levels. Data are presented as the mean ± SD. *p < 0.05, **p < 0.01, ***p < 0.001 (*n* = 3 independent experiments)

To explore the role of GDF7 in osteogenic differentiation, hADSCs were co-transfected with miR-204-5p mimics and GDF7 plasmid or co-transfected with miR-204-5p inhibitor and GDF7 siRNA. Compared with the empty vector transfection, the results showed that overexpression of GDF7 could inhibit miR-204-5p expression but promote the mRNA and protein expression of GDF7, RUNX2, ALP and OCN, whereas downregulation of GDF7 obtained opposite results (Fig. 6a, b). Recent studies proved that the AKT and P38 signalling pathways were involved in osteogenic differentiation, so we next carried out Western blotting to evaluate the protein expression levels of p-AKT, AKT, p-P38 and P38. The results suggested that overexpression of GDF7 could enhance the phosphorylation of AKT and P38 under treatment with miR-204-5p mimics, while knockdown of GDF7 reduced the phosphorylation of AKT and P38 under inhibition of miR-204-5p (Fig. 6c).

**Fig. 7:**
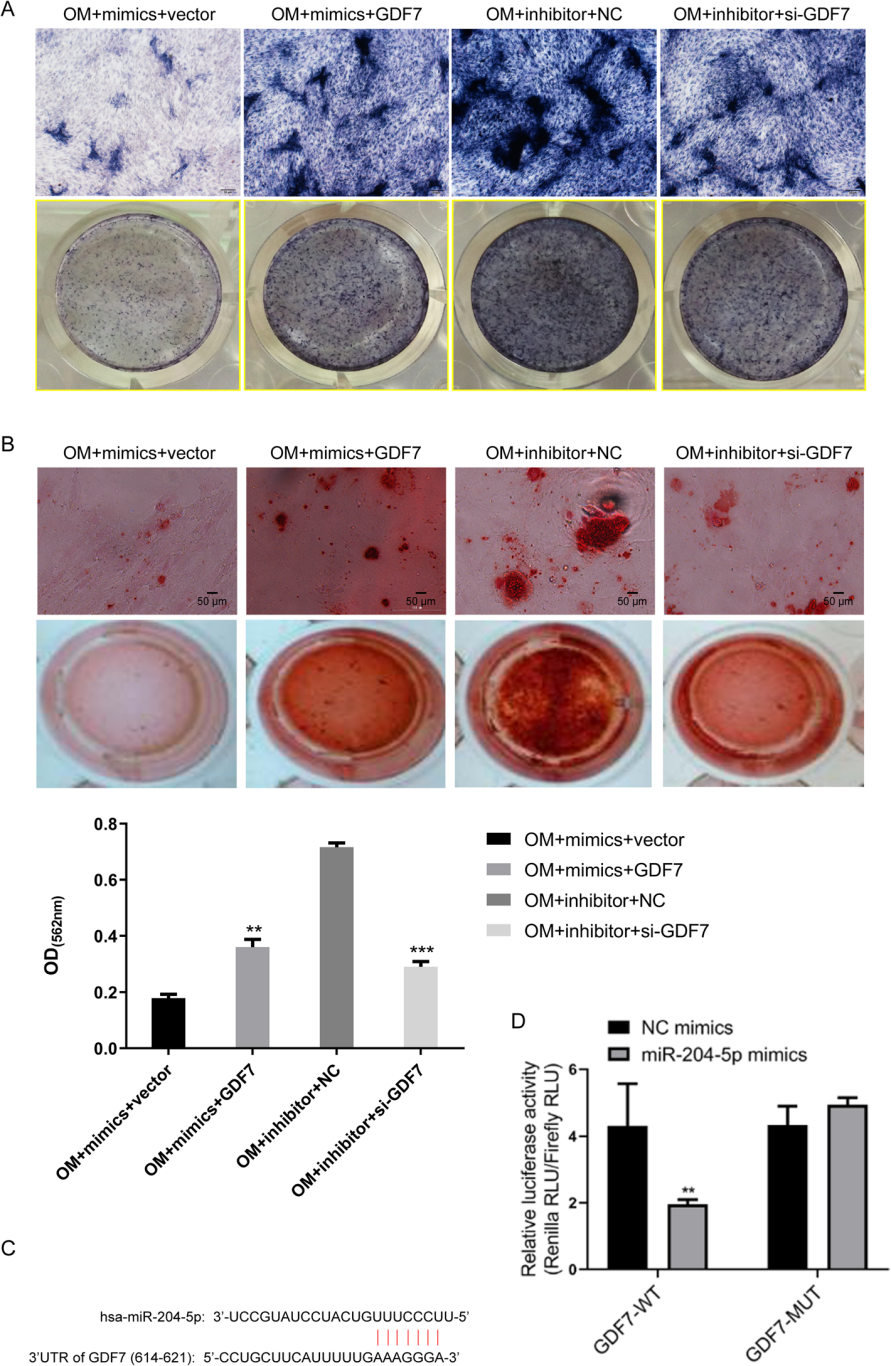
GDF7 impairs the suppressive effect of miR-204-5p on the osteogenic differentiation of hADSCs. hADSCs were co-transfected with miR-204-5p mimics and GDF7-overexpression vector or empty vector or co-transfected with miR-204-5p inhibitors and si-GDF and cultured in OM. ALP staining (**a**) and ARS (**b**) on day 14 after osteogenic induction. Quantification of ARS was shown below. **c** Predicted binding site of miR-204-5p in the 3′-UTR of GDF7 mRNA. **d** Relative luciferase activity of cells in different groups. Data are presented as the mean ± SD. *p < 0.05, **p < 0.01, ***p < 0.001 (*n* = 3 independent experiments)

Moreover, ALP staining showed that overexpression of GDF7 promoted the osteogenic differentiation of hADSCs transfected with miR-204-5p mimics, while downregulation of GDF7 suppressed this process in hADSCs transfected with miR-204-5p inhibitor (Fig. 7a). ARS showed that overexpression of GDF7 increased the extracellular mineralization of hADSCs transfected with miR-204-5p mimics, but downregulation of GDF7 inhibited the extracellular mineralization of hADSCs transfected with miR-204-5p inhibitor (Fig. 7b). Taken together, these results indicated that GDF7 impairs the suppressive effect of miR-204-5p on the osteogenic differentiation of hADSCs via the AKT and p38 signalling pathways.

Finally, we used a dual-luciferase reporter assay to verify whether *GDF7* is a potential target of miR-204-5p. We found that there is a miR-204-5p binding site in the 3′-UTR of *GDF7* (Fig. 7c). Next, we constructed a WT reporter and MUT reporter that contained a mutant 3′-UTR of GDF7 designed with mutated sequences of the miR-204-5p binding site. The results indicated that overexpression of miR-204-5p markedly decreased the luciferase activity in the WT group of GDF7 cells but not in the MUT group (Fig. 7d). This result suggested that *GDF7* is also a target of miR-204-5p.

### FOXC1, as a transcription factor, could bind to the promoter of miR-204-5p and GDF7

**Fig. 8:**
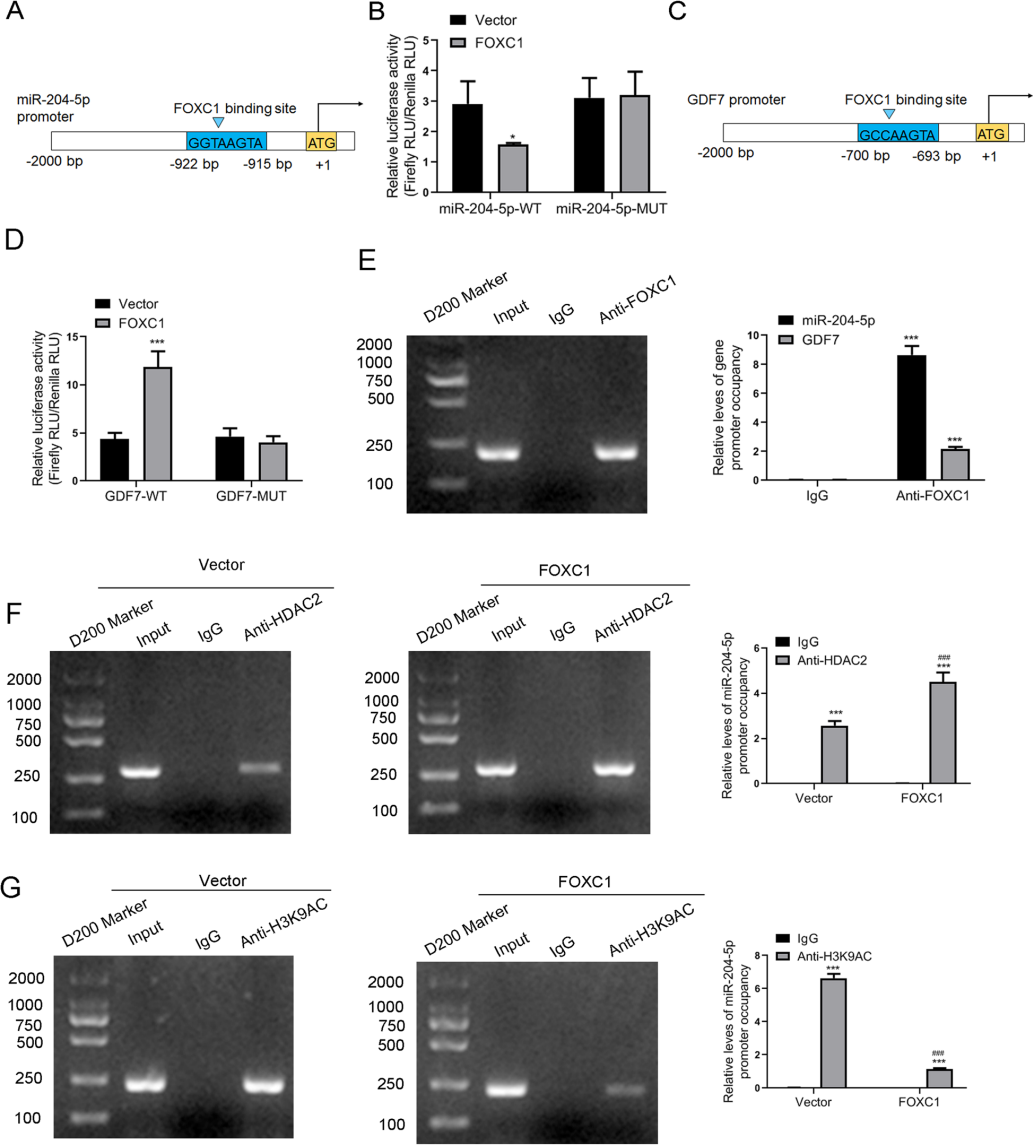
FOXC1, as a transcription factor, binds to the promoter of miR-204-5p and GDF7. **a** Predicted binding site of FOXC1 (GGTAAGTA) in the miR-204-5p promoter (− 922 to − 915 bp). **b** Relative luciferase activity of cells transfected with vector/vector-FOXC1 and miR-204-5p-WT or vector/vector-FOXC1 and miR-204-5p-MUT. Data are presented as the mean ± SD. *p < 0.05 (*n* = 3 independent experiments). **c** Predicted binding site of FOXC1 (GCCAAGTA) in the GDF7 promoter (− 700 to − 693 bp). **d** Relative luciferase activity of cells transfected with vector/vector-FOXC1 and GDF7-WT or vector/vector-FOXC1 and GDF7-MUT. Data are presented as the mean ± SD. ***p < 0.001 (*n* = 3 independent experiments). **e** The enrichment of FOXC1 in the promoter of GDF7 (left and right panel) and miR-204-5p (right panel) was measured by gel electrophoresis and ChIP assay. Normal IgG was used as a negative control. **f** The enrichment of histone deacetylase HDAC2 in the promoter of miR-204-5p was measured by gel electrophoresis and ChIP assay. **g** The enrichment of histone acetylase H3K9AC in the promoter of miR-204-5p was measured by gel electrophoresis and ChIP assay. Data are presented as the mean ± SD. ***anti-HDAC2 or anti-H3K9AC vs IgG, ^###^overexpression of FOXC1 vs vectors in HDAC2 or H3K9AC samples. ***p < 0.001, ^###^*p* < 0.001 (*n* = 3 independent experiments)

Then, we sought to explore the specific regulatory mechanism among miR-204-5p, FOXC1 and GDF7 in the osteoblast differentiation of hADSCs. We speculated that the transcription factor FOXC1 might bind to the promoters of miR-204-5p and *GDF7*. As expected, a putative binding site of FOXC1 was found in the promoters of both miR-204-5p (Fig. 8a) and *GDF7* (Fig. 8c). We generated dual-luciferase reporters containing the WT or MUT promoter of miR-204-5p and *GDF7*. The results showed that overexpression of FOXC1 decreased the luciferase activity of the miR-204-5p promoter but increased the luciferase activity of the *GDF7* promoter (Fig. 8b, d), indicating that FOXC1 regulates the promoter activity of miR-204-5p and *GDF7*. Referring to the predicted binding sites in Fig. 5a, c, gel electrophoresis and ChIP assays further confirmed that FOXC1 regulated the promoters of *GDF7* and miR-204-5p through direct binding (Fig. 8e).

To further investigate the effect of FOXC1 on the transcriptional activity of the miR-204-5p promoter, we focused on histone acetylation regulation. Putative binding sites of histone deacetylase HDAC2 and histone acetylase H3K9AC were also found in the promoter of miR-204-5p. Gel electrophoresis and ChIP assays confirmed that both HDAC2 and H3K9AC were able to bind to the promoter of miR-204-5p. Furthermore, compared with empty vector control transfection, overexpression of FOXC1 promoted the interaction between histone deacetylase HDAC2 and miR-204-5p but inhibited the interaction between histone acetylase H3K9AC and miR-204-5p (Fig. 8f, g). These results proved that FOXC1 epigenetically silences miR-204-5p transcription through interaction with HDAC2 and H3K9AC.

## Discussion

In the present study, we investigated the osteogenic differentiation of hADSCs, showing that miR-204-5p was downregulated during the osteogenic differentiation of hADSCs. Moreover, miR-204-5p can suppress the expression of FOXC1 and GDF7, and FOXC1 could reduce the expression of miR-204-5p, thus promoting the expression of GDF7 and activating the AKT and P38 signalling pathways.

Compared with other MSCs, hADSCs stand out because of their abundance in the body, less invasive extraction procedures, lower incidence rate and multi-directional differentiation potential [27]. They have become a hotspot in the field of MSCs and one of the important sources of autologous stem cell therapy [28]. In this study, we found a new chain that could accelerate the hADSC osteogenic process.

In recent years, many studies have investigated the role and mechanism of microRNAs in stem cell adipogenesis or osteogenic differentiation [10]. A previous study analysed the expression profiles of microRNAs in MSCs during osteogenic differentiation and showed that miR-22 expression was upregulated during osteogenesis but reduced during MSC adipogenic differentiation [29]. Studies have reported that miR-103-3p regulates the osteogenic differentiation of bone marrow mesenchymal cells by inhibiting the target gene Satb2 [30, 31]. Moreover, a study has shown that miR-204-5p promoted the adipogenic differentiation of hADSCs by modulating DVL3 expression and suppressing Wnt/β-catenin signalling [25]. In this study, we proved that overexpression of miR-204-5p could decrease RUNX2, ALP and OCN expression and suppress the osteogenic differentiation of hADSCs, whereas inhibition of miR-204-5p could increase RUNX2, ALP and OCN expression and promote the osteogenic differentiation of hADSCs, suggesting that miR-204-5p might be an important target miRNA for hADSC-based therapies related to bone regeneration.

FOXC1 is an important regulator of the initial stage of the osteogenesis process in the membrane and cartilage. Oshiki et al. proved that Foxc1 could inhibit adipogenic processes in stem cells, and subsequent research disclosed that FOXC1 could promote RUNX2 expression by binding to the RUNX2 promoter [32]. Moreover, FOXC1 is essential for the maintenance of mesenchymal niches. This effect may be mediated by BMP signalling because FOXC1 could activate the BMP pathway to govern hair follicle stem cell quiescence [33]. In addition, inactivation of FOXC1 could result in a dramatic reduction in mouse skull vault growth [18]. However, there are some opinions against the positive effect of FOXC1 in osteogenesis. Caddy et al. described that FOXC1 downregulates BMP4-induced osteoblast differentiation [34]. FOXC1 expression is regulated by microRNAs, such as miR-138-5p, miR-374c and miR-133 [35, 36]. Our data indicated that FOXC1 is the target gene of miR-204-5p and that miR-204-5p could suppress the expression of FOXC1. Similar to mainstream views, our data also showed that FOXC1 promotes hADSC osteogenic differentiation.

Moreover, we found that GDF7, also named BMP12, is the downstream key protein in FOXC1-mediated osteogenic differentiation of hADSCs. We found that overexpression of FOXC1 could promote GDF7 expression and induce overexpression of osteogenic key proteins, such as RUNX2, ALP and OCN, and GDF7 inactivation led to the opposite results. Although many studies have indicated that GDF7 stimulates the proliferation of tendon fibroblasts and induces tendon and ligament formation [23, 37], Shinji et al. found that GDF7 gene transfer into a rat femoral bone defect induced tendon/ligament-like tissue formation until 4 weeks after the operation, and these tissues were eventually replaced with bone-like tissue by 8 weeks after the operation, suggesting that GDF7 initiated the replacement of tendon/ligament-like tissue with bony tissue [38]. In our study, in contrast to the main opinions, we found that GDF7 also plays an important role in regulating osteoblast differentiation and subsequent bone formation. The diverse conclusions may be due to the different species, and the lack of animal experiments might limit our insight.

Interestingly, we also found that GDF7 is a target gene of miR-204-5p and that miR-204-5p could suppress the expression of GDF7. In other words, miR-204-5p could downregulate both FOXC1 and GDF7 to inhibit the osteogenesis of hADSCs. To verify the specific regulatory mechanism among miR-204-5p, FOXC1 and GDF7 in the osteoblast differentiation of hADSCs, we performed dual-luciferase reporter assays and ChIP assays, and we found that the transcription factor FOXC1 could bind to the promoters of miR-204-5p and *GDF7* and enhance the translation of *GDF7*, indicating that FOXC1 functioned upstream of GDF7. However, whether FOXC1 expression may be altered by GDF7, or feedback loop might exist between these proteins, should be further determined. To our surprise, our results suggested that overexpression of FOXC1 could reverse the inhibition of hADSC osteogenic differentiation by downregulating miR-204-5p, while downregulation of FOXC1 could weaken the inhibition of miR-204-5p, thus attenuating the promotion of osteogenic differentiation by the miR-204-5p inhibitor. To further investigate the regulatory mechanism between FOXC1 and miR-204-5p, we explored the production of miR-204-5p and found that FOXC1 can promote the deacetylation of the promoter region of miR-204-5p.

The transcription of miRNA is mainly performed by RNA polymerase II, and the structure of the primary transcript pri-miRNA contains the same 7-methylguanosine cap and poly(A) tail as the primary transcript of the gene encoding the protein. In addition, miRNAs are also regulated by epigenetic modifications such as DNA methylation and histone acetylation. Research has suggested that approximately 5–10% of mammalian miRNAs are regulated by epigenetics [39]. For example, the correct expression of miR-148a, miR-34b/c, miR-9 and let-7a-3 depends on the methylation status and DNMT1, DNMT3b or other DNA methylase activities [39]. Saito et al. found that when DNA demethylation reagents and histone deacetylation inhibitors were used to treat human T24 bladder cancer cells, approximately 5% of miRNAs were upregulated by more than 3 times, especially miR-127 in CpG islands [40]. This further illustrated that the regulation of miRNA genes by epigenetic modification is in their promoter regions and can be activated by inhibiting DNA methylation and histone acetylation. However, low histone acetylation in special promoter regions often leads to gene silencing [41]. In our study, we investigated the effect of FOXC1 on the transcriptional activity of the miR-204-5p promoter by ChIP assay. The results showed that FOXC1 could weaken H3K9AC acetylation, promote HDAC2 deacetylation of the miR-204-5p promoter and reduce the transcriptional activity of miR-204-5p during osteogenic differentiation.

To date, studies have confirmed that the activation of p38 MAPK and AKT signalling pathways is the crucial stimulus for the osteogenic differentiation of hADSCs [42–44]. Our data confirmed that GDF7 overexpression can promote the phosphorylation of P38 and AKT, thus promoting the P38 and AKT signalling pathways. Moreover, this study showed that overexpression of GDF7 reversed the inhibition of osteogenic differentiation of hADSCs by miR-204-5p mimics. Additionally, FOXC1 promoted the transcriptional activity of *GDF7* by inhibiting H3K9AC acetylation and promoting HDAC2 deacetylation of the miR-204-5p promoter during osteogenic differentiation.

Consistent with previous studies, we also found that miR-204-5p, FOXC1 and GDF7 play important roles during osteogenic differentiation, and we further elucidated the underlying mechanism of the regulatory network between these factors for the first time.

**Fig. 9:**
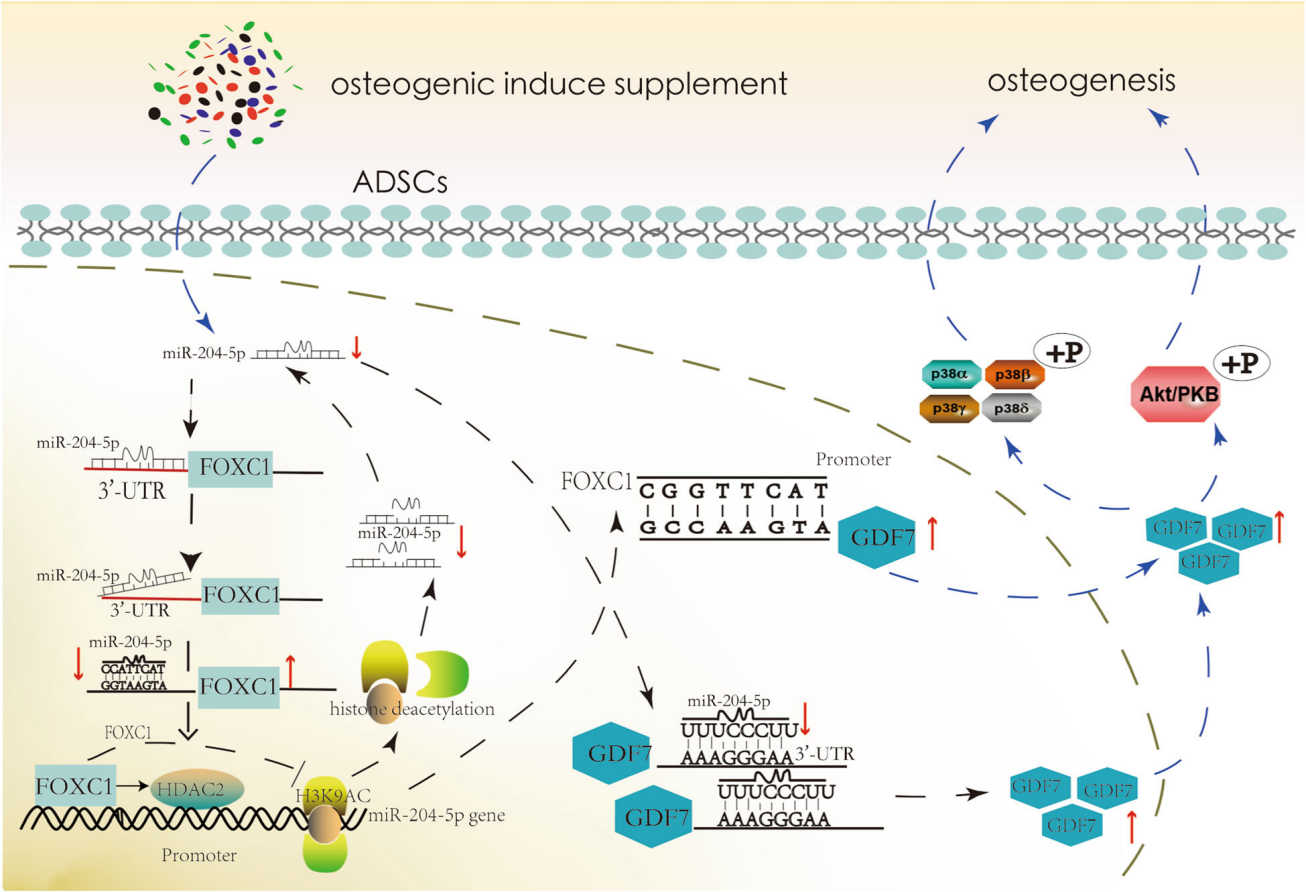
The mechanism of the miR-204-5p/FOXC1/GDF7 regulatory axis in the osteogenic differentiation of hADSCs

We summarized the mechanism of the miR-204-5p/FOXC1/GDF7 regulatory axis during osteogenic differentiation as shown in Fig. 9. miR-204-5p is downregulated during the osteogenic differentiation of hADSCs. Moreover, miR-204-5p can bind to the 3′-UTR region of *FOXC1* and *GDF7* and suppress the expression of FOXC1 and GDF7. FOXC1 bound to the promoters of miR-204-5p and *GDF7*, promoted the deacetylation of miR-204-5p and reduced the expression of miR-204-5p, thus promoting the expression of GDF7, which induced hADSC osteogenesis by activating the AKT and P38 signalling pathways. Finally, the FOXC1/GDF7/miR-204-5p axis formed a feedback loop, which plays an important role in the balance of osteogenic differentiation. The specific mechanisms of downstream signalling pathways of GDF7 and whether FOXC1 can affect the methylation of the miR-204-5p promoter will be our future research focuses.

Modification of the miRNAs that mediate osteogenic differentiation has become an approach to optimize the osteogenic potential of hADSCs. One strategy is to either expose the cells to inhibitors of these miRNAs or to engineer cells that lack expression of these miRNAs. Another is to target the upstream factors that regulate these miRNAs. Since we found that miR-204-5p was downregulated during the osteogenic differentiation of hADSCs and that FOXC1 could bind to the promoter of miR-204-5p, functioning as an upstream mediator of miR-204-5p, modification of the expression of miR-204-5p of FOXC1 could be an efficient approach in bone tissue engineering and bone healing therapies.

## Conclusion

Our results demonstrated that the miR-204-5p/FOXC1/GDF7 axis regulates the osteogenic differentiation of hADSCs via the AKT and p38 signalling pathways. This study further revealed the regulatory mechanism of hADSC differentiation from the perspective of miRNA regulation.

## Data Availability

Please contact the authors for data requests.

## Abbreviations

hADSCs: Human adipose-derived stem cells
FOXC1: Forkhead box C1
GDF7: Growth differentiation factor 7
BMP12: Bone morphogenetic protein 12
CDMP3: Cartilage-derived morphogenetic protein 3
miRNA: MicroRNA
MSCs: Mesenchymal stem cells
TGF-β: Transforming growth factor beta
PBS: Phosphate-buffered saline
DMEM F12: Dulbecco’s modified Eagle’s medium/Nutrient F-12
FBS: Foetal bovine serum
PM: Proliferation medium
OM: Osteogenic differentiation medium
ALP: Alkaline phosphatase
ARS: Alizarin red staining
DAPI: 40,6-Diamidino-2-phenylindole
WT: Wild-type
MUT: Mutant-type
ChIP: Chromatin immunoprecipitation
IgG: Immunoglobulin G
3′-UTR: 3′ untranslated region
CXCL: CXC chemokine ligand
CAR cells: CXCL 12-abundant reticular cells
